# Correction: HER2 inhibition increases non-muscle myosin IIA to promote tumorigenesis in HER2+ breast cancers

**DOI:** 10.1371/journal.pone.0315773

**Published:** 2024-12-10

**Authors:** The PLOS ONE Staff

## Notice of Republication

This article was republished on November 22, 2024, to correct an error in Fig 3 that was introduced during the typesetting process. The publisher apologizes for the errors. Please download this article again to view the correct version. The originally published, uncorrected article and the republished, corrected articles are provided here for reference.

## Supporting information

S1 File Originally published, uncorrected article. (PDF)

S2 File Republished, corrected article. (PDF)
