# Supplementary figures and images for: HER2 inhibition increases non-muscle myosin IIA to promote tumorigenesis in HER2+ breast cancers
Source: PLoS One. 2023 May 18;18(5):e0285251. doi: 10.1371/journal.pone.0285251 (PMC10194889; doi:10.1371/journal.pone.0285251)

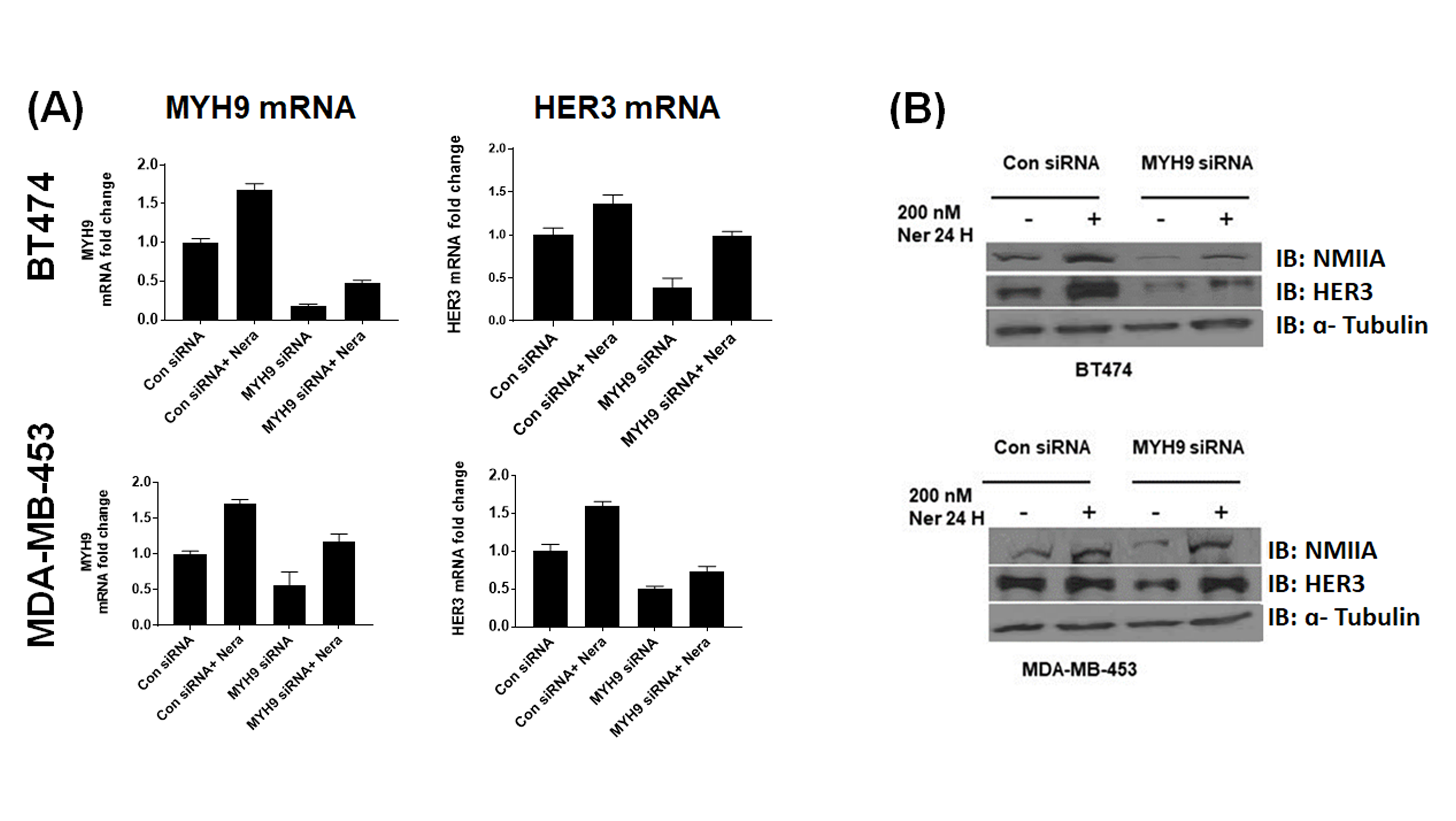

Supplement: S1 Fig — BT474 and MDA-MB-453 were seeded in 60 mm plates (2x 10^6^ cells/plate), and forward and reverse transfection were performed using siRNA targeting MYH9 or scrambled sequence (control) and lipofectamine RNAmax mixture for 48 hours. (A) BT474 and MDA-MB-453 cells were then treated with 200 nM neratinib or DMSO for 24 hours. RNA was isolated and MYH9 and HER3 RNA levels determined using real time PCR. (B) BT474 and MDAMB-453 cells were treated with neratinib (200 nM) for 0–24 hours. Cells were lysed and products analyzed by 10% SDS-PAGE followed by probing immunoblots with antibodies for NMIIA, HER3 and α-tubulin. (TIF) [file pone.0285251.s001.tif]

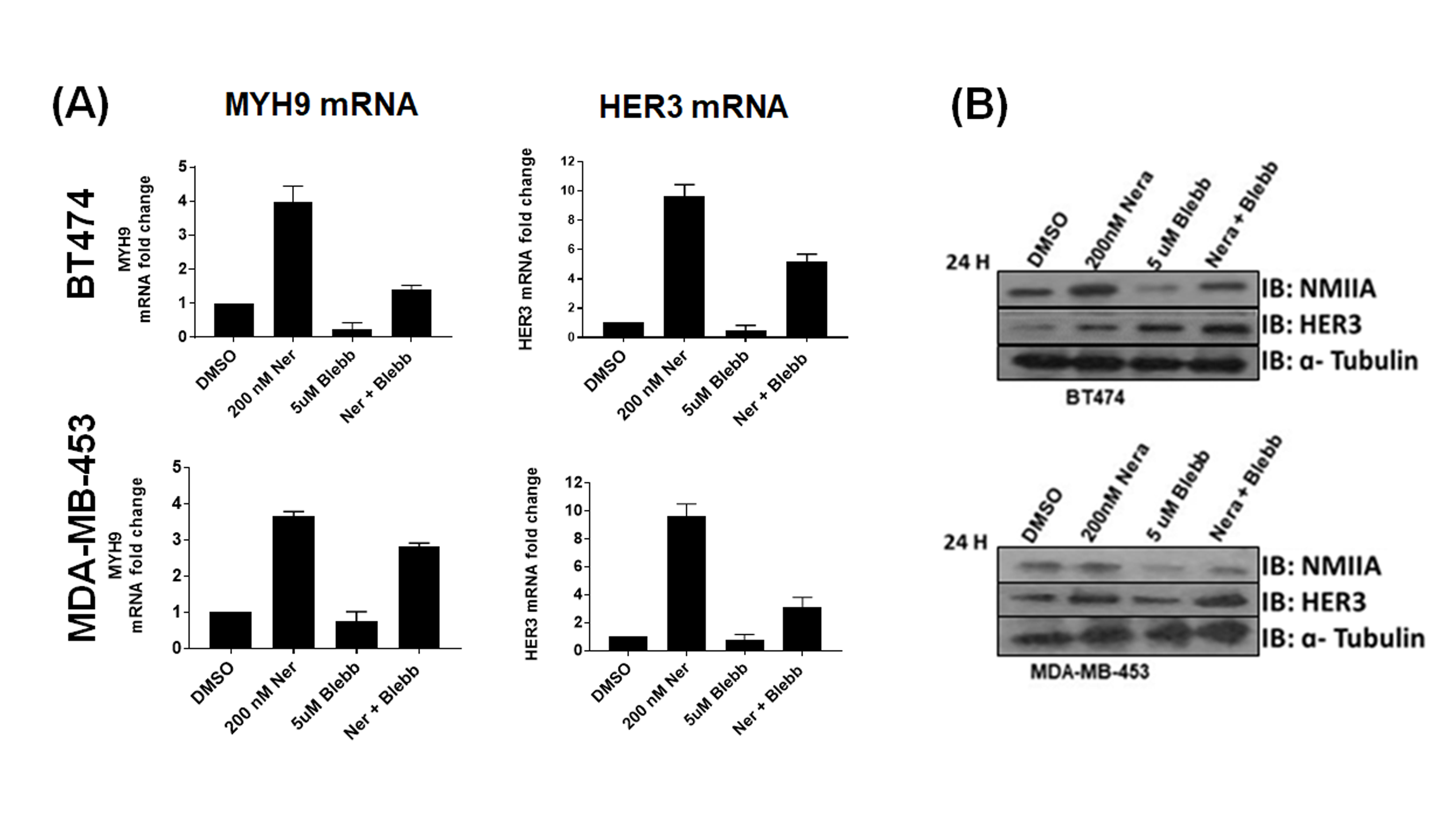

Supplement: S2 Fig — (A) BT474 and MDA-MB-453 cells were treated with DMSO, neratinib, blebbistatin, and combination therapy of neratinib and blebbistatin for 24 hours. RNA was isolated and MYH9 and HER3 RNA levels determined using real time PCR. Graphs were blotted in Prism 7 (GraphPad) (B) BT474 and MDA-MB-453 cells were treated with DMSO, neratinib, blebbistatin, and combination therapy of neratinib and blebbistatin for 24 hours. Cells were lysed in RIPA buffer and the products were analyzed by 10% SDS-PAGE followed by probing immunoblots with antibodies against NMIIA, HER3, and α-tubulin. (TIF) [file pone.0285251.s002.tif]

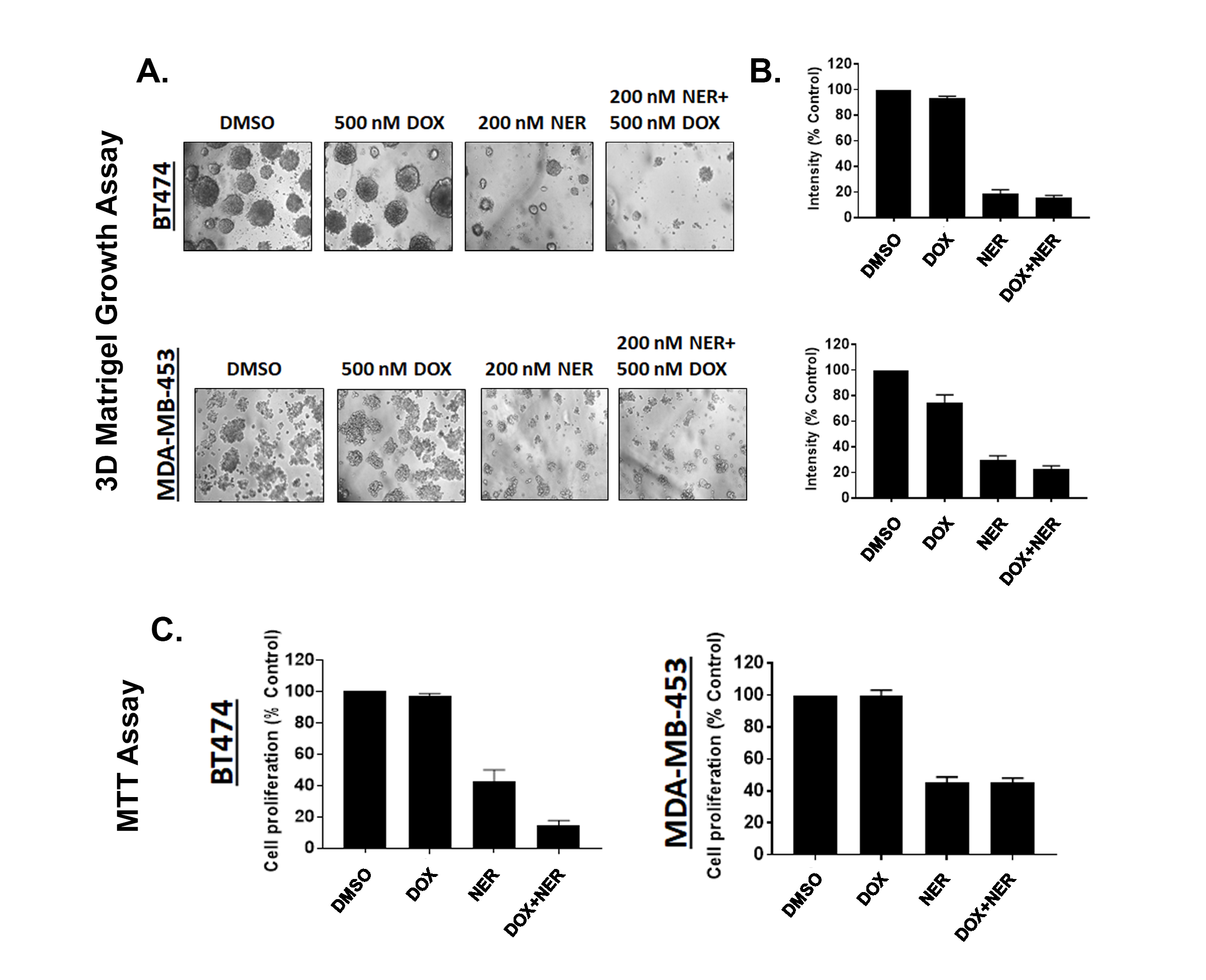

Supplement: S3 Fig — (A) BT474 and MDA-MB-453 cells transduced with non-targeting control shRNA were seeded on a basement membrane of matrigel at a density of 2 × 10^4^ cells/well. Cells treated with vehicle (DMSO), doxycycline (500 nM)/48 h, neratinib (200 nM) once, and combination. Phase contrast images of acini for cell lines were captured at 10× magnification and the average size of each cellular structure was quantified and expressed as mean of areas ± SEM, n = 5 random fields (B). (C) BT474 and MDA-MB-453 cells transduced with a doxycycline inducible shRNA targeting sh-control (shCTRL) were treated with doxycycline (500 nM) and neratinib (200 nM) for 72 hr. After 72 hr, the media containing the drug was replaced with 5 mg/mL MTT dissolved in cell line specific media and incubated for 4 hr. After 4 hr the media was aspirated, and crystals were dissolved with isopropanol. The bar graphs are represented as mean; Error bars: SEM, n = 3. (TIF) [file pone.0285251.s003.tif]

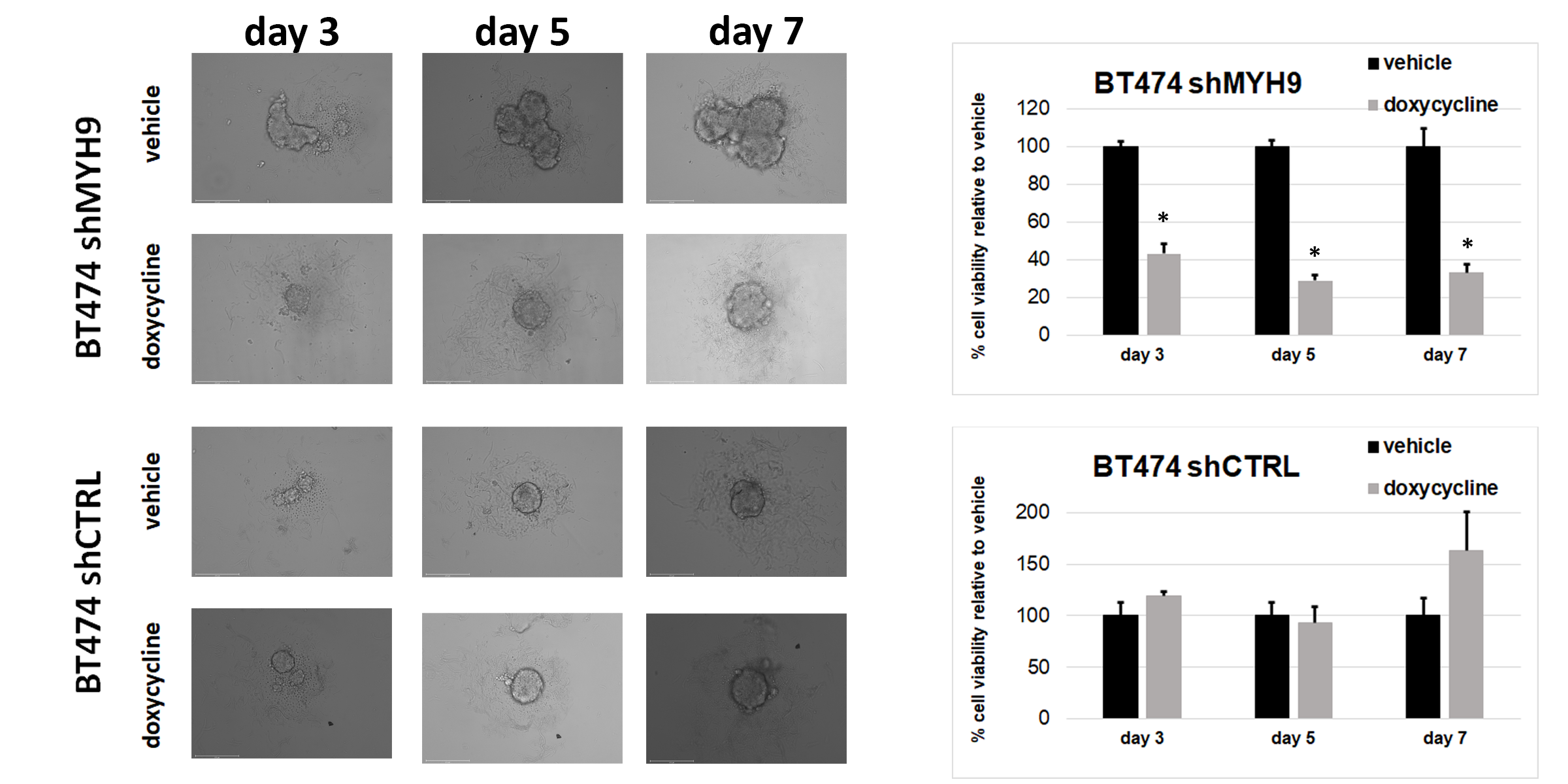

Supplement: S4 Fig — 1000 cells expressing shRNA targeting MYH9 or cell expressing non-targeting control (shCTRL) were plated in 96-well Ultra-Low Attachment plates. Cells were treated upon plating and every 48 hours with 500 nM doxycycline or vehicle. Images of spheroids were taken 10x magnification (left panel). PrestoBlue reagent was added on days 3, 5, and 7 to assess cell viability. Data shown is normalized to the values for vehicle treatment. The bar graphs are represented as mean; Error bars: SEM, n = 3. * p <0.05 for comparison to vehicle (right panel). (TIF) [file pone.0285251.s004.tif]

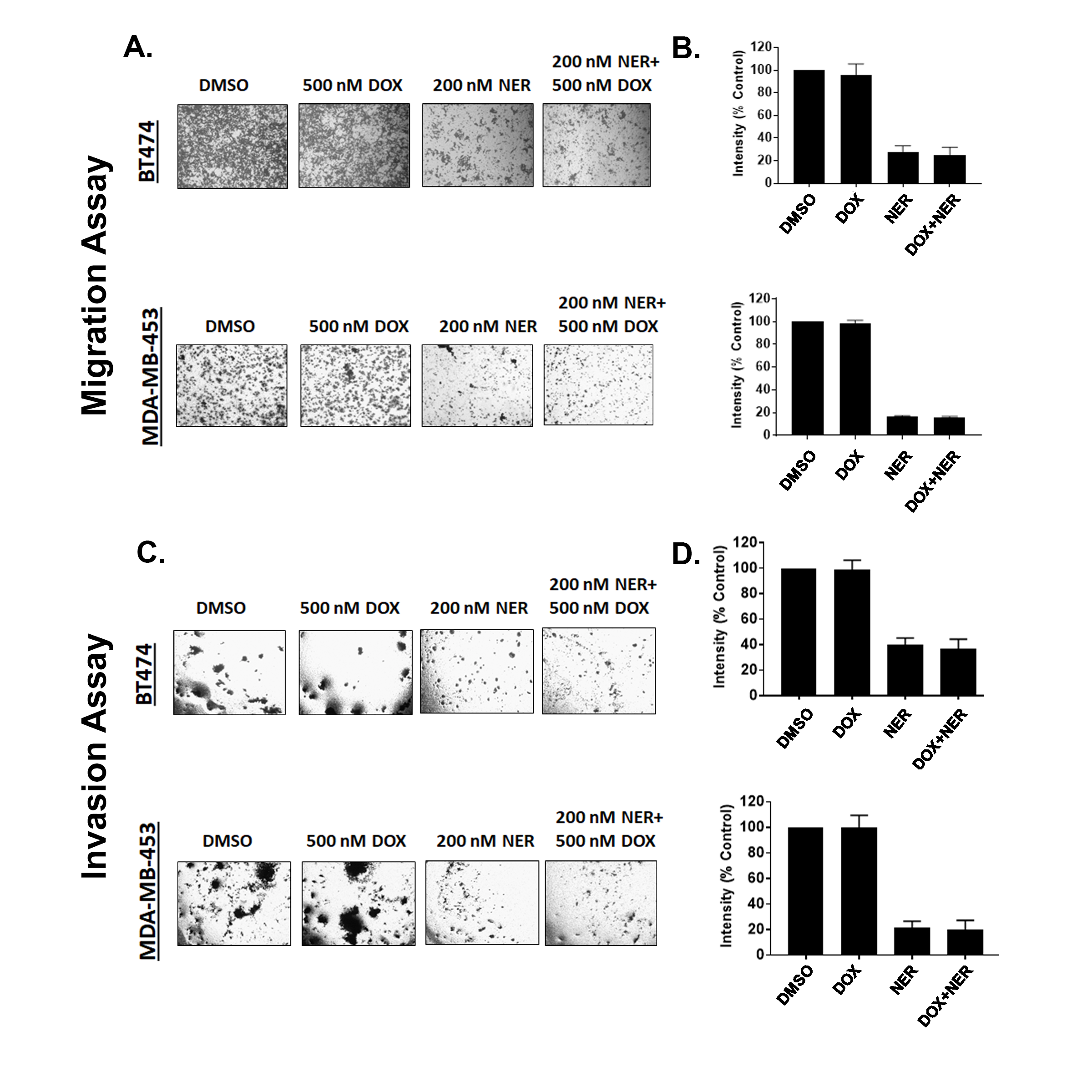

Supplement: S5 Fig — (A) BT474 and MDA-MB-453 (5 × 10^4^ cells/well) stable cells transduced with non-targeting control shRNA were seeded in six well plates and treated with vehicle (DMSO), doxycycline (500 nM), neratinib (200 nM) and indicated combination in serum free media for 24 h. Post 24 hr treatment, these treated cells (2 × 10^4^ cells/well) were added to the upper chamber of transwell plates with 10% FBS as chemoattractant in the lower chamber. After 24h, the migrated cells were stained and captured. (B) The intensities of the migrated cells were measured using ImageJ and expressed as mean of % control and represented as bar graph. Error bars: SEM, n = 4 (C) Invasion assays were performed where each chamber was coated with 20 <l matrigel mixed with 80 <l serum free media and incubated at 37° for 30 mins. Stable cells (BT474 and MDA-MB-453) expressing non-targeting control shRNA were seeded at a density of 5 × 10^4^ cells/well and treated and incubated as above. After 24h, the invaded cells were stained and photographed (D) The intensities of the invaded cells were measured using ImageJ and expressed as mean % of control. Error bars: SEM, n = 3. (TIF) [file pone.0285251.s005.tif]
