# Supplementary material for: HER2 inhibition increases non-muscle myosin IIA to promote tumorigenesis in HER2+ breast cancers
Source: PLoS One. 2023 May 18;18(5):e0285251. doi: 10.1371/journal.pone.0285251 (PMC10194889; doi:10.1371/journal.pone.0285251)

Whole blot images of HER3, and NMIIA in BT474 cells from Figure 1B and MDA-MB-453 cells from Figure 1C

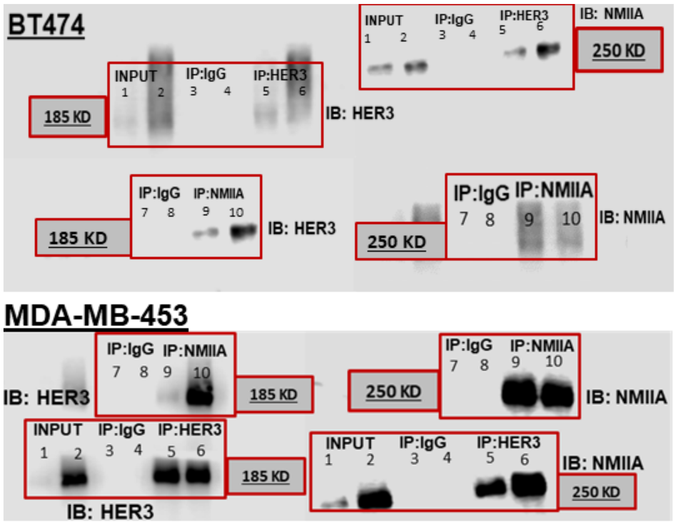

1) DMSO
2) 200 nM Ner (24H) } INPUT
3) DMSO
4) 200 nM Ner (24H) } IP:IgG
5) DMSO
6) 200 nM Ner (24H) } IP:HER3
7) DMSO
8) 200 nM Ner (24H) } IP:IgG
9) DMSO
10) 200 nM Ner (24H) } IP:NMIIA

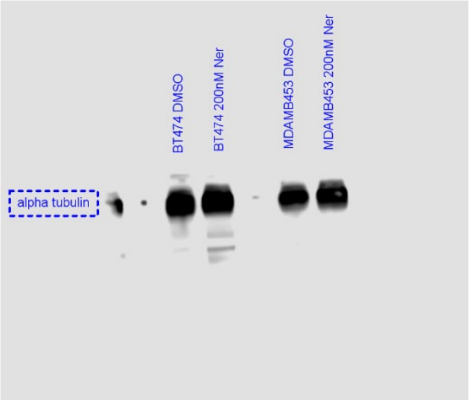

Whole blot images of P-HER2, HER2, NMIIA/ HER3/ α-Tubulin in BT474 and MDA-MB-453 cells from Figure 3B

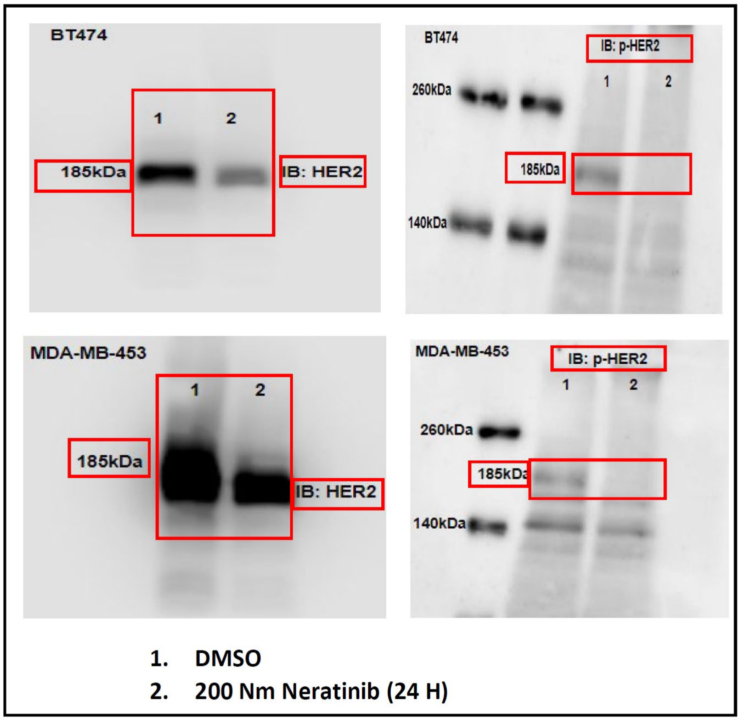

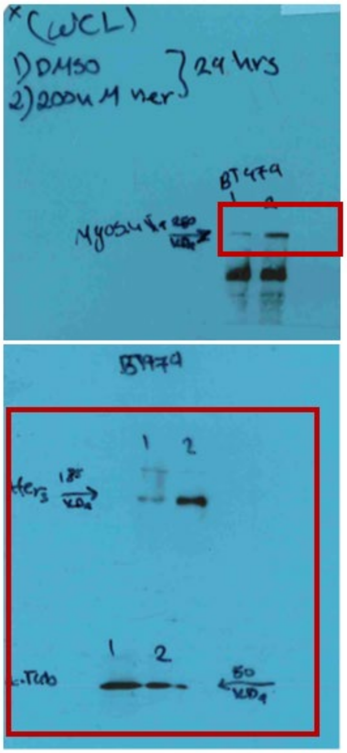

Figure 3 B. BT474

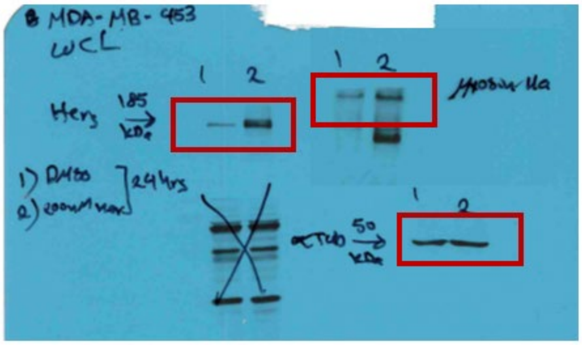

Figure 3 B. MDA-MB-453

Whole blot images of NMIIA/ α-Tubulin in BT474 and MDA-MB-453 cells from Figure 4A

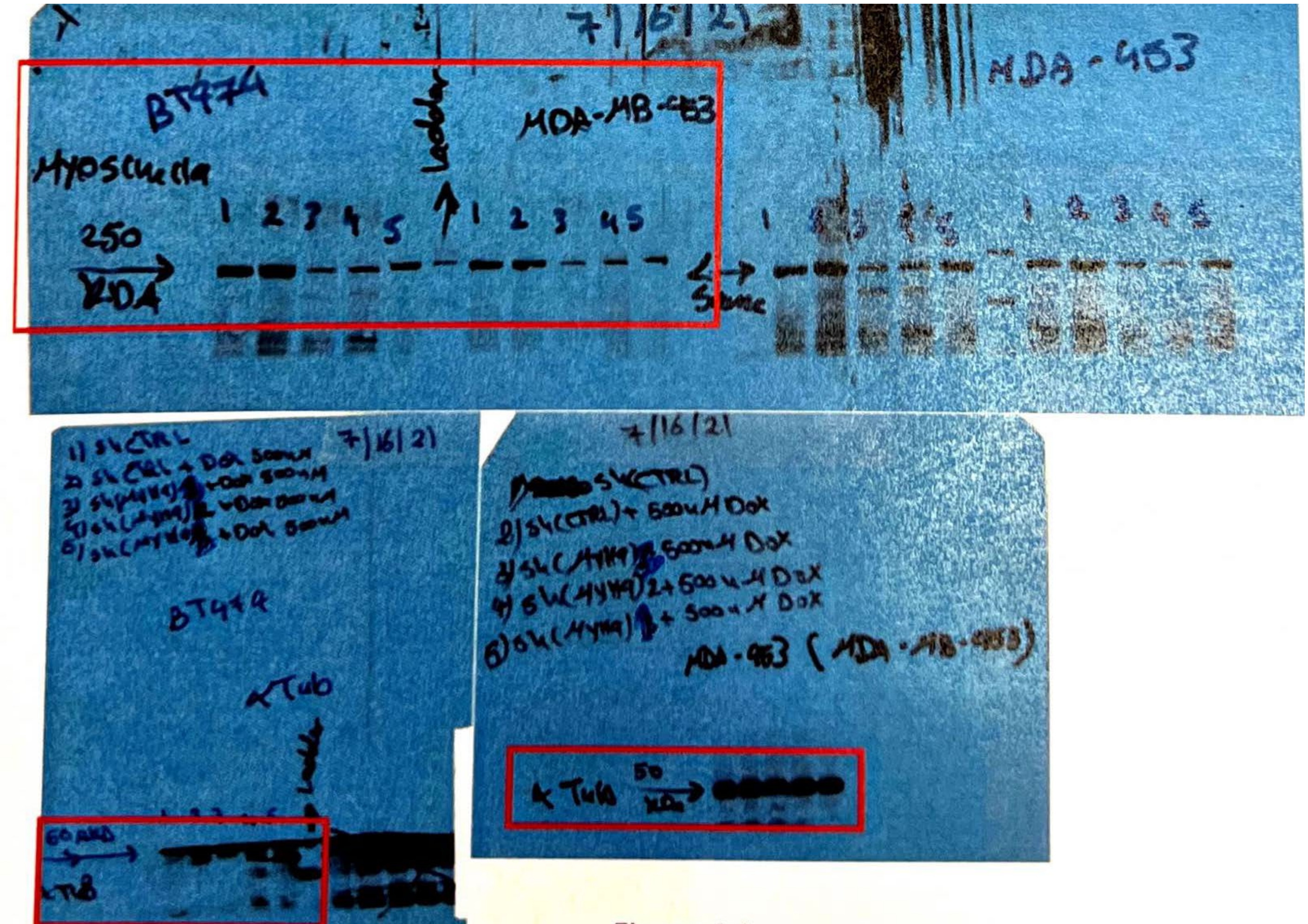

Figure 4 A.

Whole blot images of NMIIA/ p-HER3 (Y1289)/ HER3/ p-AKT (T308)/ AKT/ p-ERK1/2/ ERK1/2/ α-Tubulin in BT474 and MDA-MB-453 cells from Figure 4B

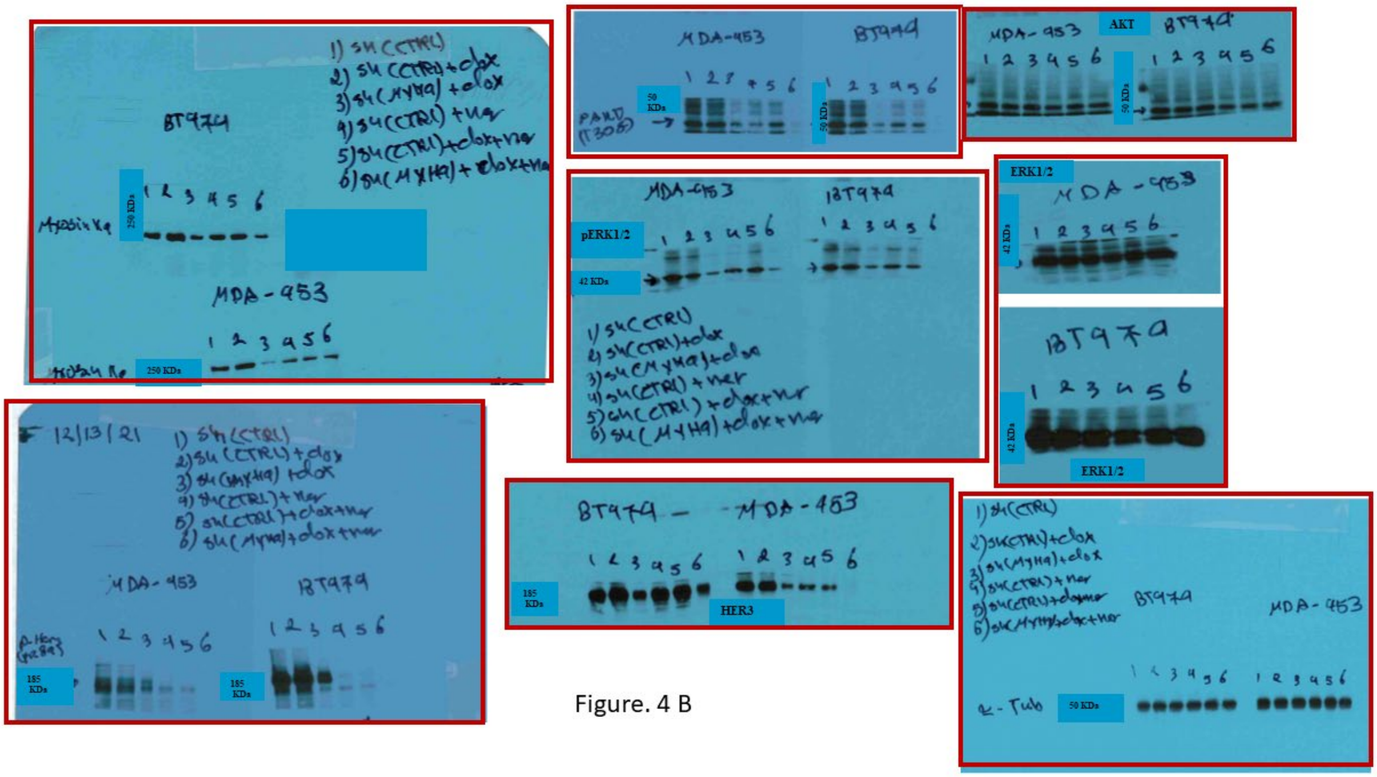

Figure. 4 B

Whole blot images of NMIIA/ HER3/ α-Tubulin in BT474 and MDA-MB-453 cells from Figure S1 B

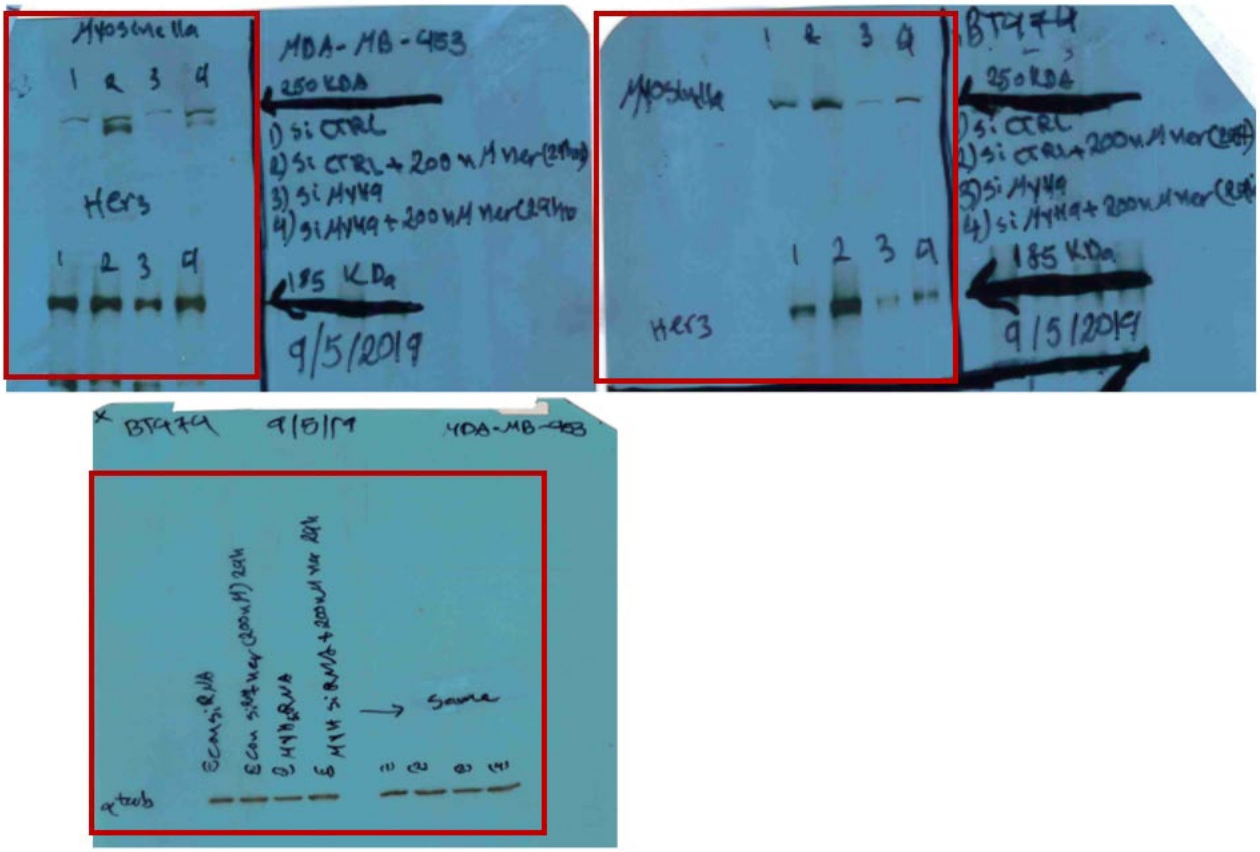

Whole blot images of NMIIA/ HER3/ α-Tubulin in BT474 and MDA-MB-453 cells from Figure S2 B

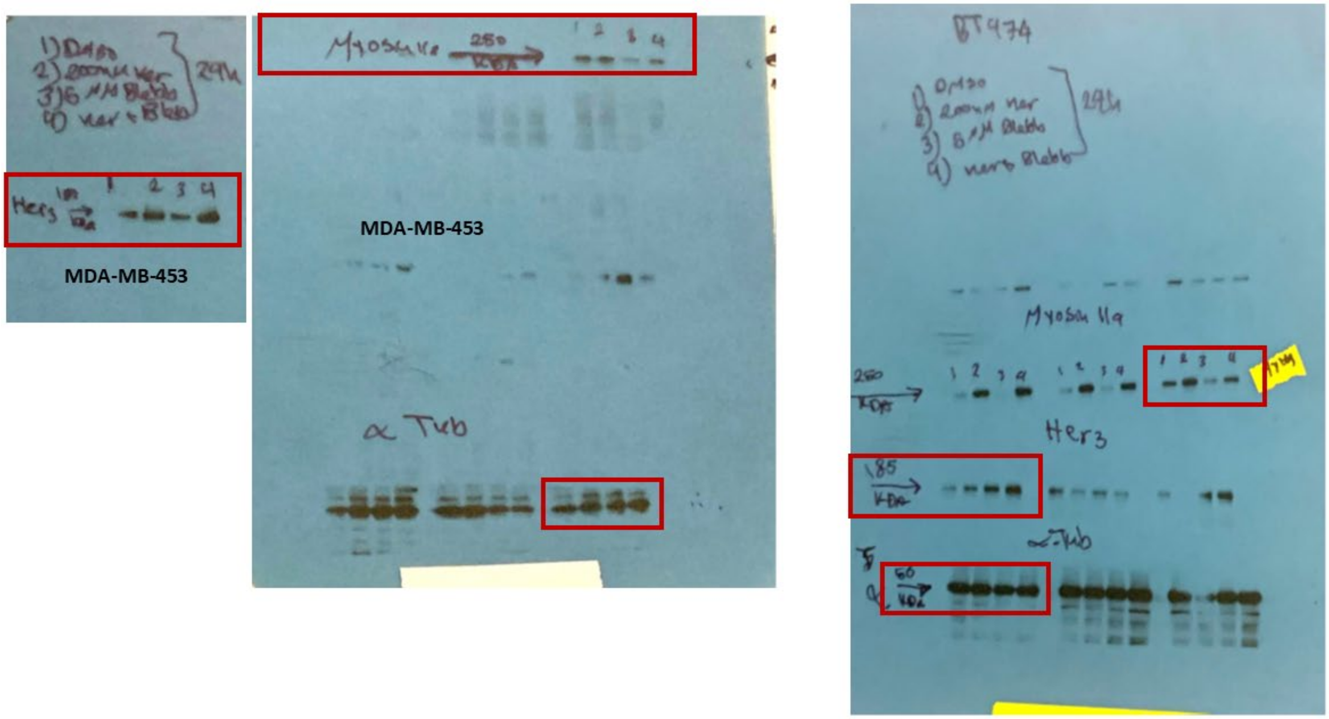

Supplement: S1 Raw images — (PDF) [file pone.0285251.s006.pdf]
